# Supplementary material for: Support for a clade of Placozoa and Cnidaria in genes with minimal compositional bias
Source: eLife. 2018 Oct 30;7:e36278. doi: 10.7554/eLife.36278 (PMC6277202; doi:10.7554/eLife.36278)
Supplement: Transparent reporting form [file elife-36278-transrepform.docx]

***eLife’s* transparent reporting form**

We encourage authors to provide detailed information *within their submission* to facilitate the interpretation and replication of experiments. Authors can upload supporting documentation to indicate the use of appropriate reporting guidelines for health-related research (see [EQUATOR Network](http://www.equator-network.org/%20)), life science research (see the [BioSharing Information Resource](https://biosharing.org/)), or the [ARRIVE guidelines](http://www.plosbiology.org/article/info:doi/10.1371/journal.pbio.1000412) for reporting work involving animal research. Where applicable, authors should refer to any relevant reporting standards documents in this form.

If you have any questions, please consult our Journal Policies and/or contact us: [editorial@elifesciences.org](mailto:editorial@elifesciences.org).

**Sample-size estimation**

- You should state whether an appropriate sample size was computed when the study was being designed
- You should state the statistical method of sample size computation and any required assumptions
- If no explicit power analysis was used, you should describe how you decided what sample (replicate) size (number) to use

Please outline where this information can be found within the submission (e.g., sections or figure legends), or explain why this information doesn’t apply to your submission:

Our study is purely phylogenetic and contains no experiments per se, and therefore conventional statistical concepts of replication and sample size do not apply.

**Replicates**

- You should report how often each experiment was performed
- You should include a definition of biological versus technical replication
- The data obtained should be provided and sufficient information should be provided to indicate the number of independent biological and/or technical replicates
- If you encountered any outliers, you should describe how these were handled
- Criteria for exclusion/inclusion of data should be clearly stated
- High-throughput sequence data should be uploaded before submission, with a private link for reviewers provided (these are available from both GEO and ArrayExpress)

Please outline where this information can be found within the submission (e.g., sections or figure legends), or explain why this information doesn’t apply to your submission:

We clearly state chain lengths and bootstrap resampling replicates for nodal support estimation within the “phylogenetic inference” portion of the Methods section.

**Statistical reporting**

- Statistical analysis methods should be described and justified
- Raw data should be presented in figures whenever informative to do so (typically when N per group is less than 10)
- For each experiment, you should identify the statistical tests used, exact values of N, definitions of center, methods of multiple test correction, and dispersion and precision measures (e.g., mean, median, SD, SEM, confidence intervals; and, for the major substantive results, a measure of effect size (e.g., Pearson's r, Cohen's d)
- Report exact p-values wherever possible alongside the summary statistics and 95% confidence intervals. These should be reported for all key questions and not only when the p-value is less than 0.05.

Please outline where this information can be found within the submission (e.g., sections or figure legends), or explain why this information doesn’t apply to your submission:

Statistical tests used to investigate compositional bias are described under the “Tests of compositional heterogeneity” header in the Methods section. Original posterior predictive test output files and tsv files defining outcomes of the per-gene compositional heterogeneity test are included in the DataDryad accession (see below)

(For large datasets, or papers with a very large number of statistical tests, you may upload a single table file with tests, Ns, etc., with reference to sections in the manuscript.)

**Group allocation**

- Indicate how samples were allocated into experimental groups (in the case of clinical studies, please specify allocation to treatment method); if randomization was used, please also state if restricted randomization was applied
- Indicate if masking was used during group allocation, data collection and/or data analysis

Please outline where this information can be found within the submission (e.g., sections or figure legends), or explain why this information doesn’t apply to your submission:

This paper does not report the outcomes of experiments.

**Additional data files (“source data”)**

- We encourage you to upload relevant additional data files, such as numerical data that are represented as a graph in a figure, or as a summary table
- Where provided, these should be in the most useful format, and they can be uploaded as “Source data” files linked to a main figure or table
- Include model definition files including the full list of parameters used
- Include code used for data analysis (e.g., R, MatLab)
- Avoid stating that data files are “available upon request”

Please indicate the figures or tables for which source data files have been provided:

All original peptide data, orthogroups, orthologue alignments, supermatrices, phylogenetic and statistical test outputs have been accessioned at DataDryad under DOI: https://doi.org/10.5061/dryad.6cm1166. Original RNA reads for the 3 newly reported Placozoa genomes are also included in this accession under a separate tarball, and will be separately uploaded to NCBI’s SRA; all other original sequence data reported in this paper are already SRA accessioned as described in the Methods secton under header “Predicting proteomes from transcriptome and genome assemblies”. A README file explains the directory structure within the tarball, and describes which original files were used to produce which figures within the manuscript. Some simple Python helper scripts are also included within this tarball within appropriate subdirectories; these are not licensed and may be reused and modified without restriction. The full PhyloBayes .chains files saved during this study from amino acid level analyses have been uploaded as a separate accession to Zenodo at https://doi.org/10.5281/zenodo.1197272, due to the very large size of these files.
